# Referral to treatment times in the National Health Service of England: A five-year analysis of the impact of the COVID-19 Pandemic and socioeconomic deprivation and future implications for Ear, Nose and Throat service delivery

**DOI:** 10.1371/journal.pone.0346596

**Published:** 2026-04-06

**Authors:** Dimitrios Spinos, Thomas Beech, Jonathan Lee, Christopher Coulson, Laura French, Sheila Greenfield, Ian Litchfield, Paul Nankivell, Richard Allen, Jameel Muzaffar

**Affiliations:** 1 Department of Cancer and Genomic Sciences, University of Birmingham, Birmingham, United Kingdom; 2 Department of Ear, Nose and Throat Surgery, University Hospitals Birmingham, Birmingham, United Kingdom; 3 Department of Ear, Nose and Throat Surgery, University Hospitals Coventry and Warwickshire Foundation Trust, United Kingdom; 4 NHS Derby and Derbyshire Integrated Care Board; 5 Department of Applied Health Sciences, University of Birmingham, Birmingham, United Kingdom; 6 The Open University, Patient Partner, United Kingdom; University College London, UNITED KINGDOM OF GREAT BRITAIN AND NORTHERN IRELAND

## Abstract

**Introduction:**

Referral-to-Treatment (RTT) waiting times are a critical indicator of healthcare system efficiency and equity. In England, Ear, Nose and Throat (ENT) services are among the most affected, with over half of patients exceeding the 18-week National Health Service (NHS) target. The COVID-19 pandemic exacerbated pre-existing delays and regional disparities. This study evaluates RTT waiting time trends over five years and investigates the influence of socioeconomic deprivation and ethnicity on these patterns.

**Methods:**

Data from the NHS covering August 2019–August 2024 were analysed across regions and Integrated Care Boards (ICBs). Population, ethnicity, and deprivation data were incorporated from governmental datasets. Descriptive and inferential statistics, including Spearman correlation, were used to assess associations between RTT metrics and deprivation, ethnicity, and regional variation.

**Results:**

The number of patients on ENT waiting lists doubled nationally, with a 22% patient population increase. The Midlands showed the highest growth in incomplete pathways. Despite short-term improvements post-COVID-19, national RTT compliance and median waiting times worsened. ICB-level data from 2021 onward revealed wide performance variation, but no statistically significant association to deprivation metrics, proportion of ethnic minorities or population differences. Trends in change over time also demonstrated no significant monotonic relationships.

**Discussion:**

Despite national evidence linking deprivation and ethnicity to healthcare inequalities, these associations were not observed at ICB level when it comes to median wait times, suggesting potential masking effects of geographic aggregation or unmeasured confounders. New policies promise opportunities for increase in the capacity of service delivery, but further investigations are required to safeguard the equitable access to such services.

**Conclusion:**

ENT services remain under significant strain, with limited recovery post-pandemic. Community-based NHS care models present new opportunities for ENT’s largely non-surgical management pathways.

## Introduction

Healthcare waiting times are a critical metric for system efficiency, patient satisfaction, and equitable access within and between health systems [1–3]. Long Referral-To-Treatment (RTT) waiting times have long been recognised as a barrier to effective healthcare delivery, particularly within universal healthcare systems, such as the United Kingdom’s (UK) National Health Service (NHS) [4]. Prolonged delays not only exacerbate health disparities but also worsen patient outcomes and increase costs associated with advanced treatments required due to delayed care [5]. Recent reports have emphasised the role of systemic inequities, such as socioeconomic deprivation, as one of the largest contributors to these delays [6,7]. The COVID-19 pandemic led to dramatic increases in waiting list challenges, and although some degree of recovery on the waiting times has been observed, there remains significant variation of these rates across the country [8].

In 2009 the NHS Constitution set the standard of 92% of patients waiting no longer than 18 weeks from referral to treatment, which continued to be the standard in the latest version in 2012, as well as in all subsequent updates and amendments [9–11]. In December 2024, 7.5 million patients in England were waiting for an appointment, with more than two fifths waiting beyond the NHS standard [12]. That proportion varies by specialty, with Ear, Nose and Throat (ENT) demonstrating the worst waiting times with around half of the patients exceeding the 18-week target [9]. ENT outpatient clinic appointments have a lower conversion rate compared to other surgical specialties, such as orthopaedics, and patients are more commonly managed with advice, medications, and then discharged [13–16], indicating that they can often be effectively managed remotely.

In January 2025, NHS England released their new strategy on reforming elective care for patients, highlighting the importance of a paradigm shift away from hospital care [17]. ENT was identified as one of the five specialties that need to set clear targets and transform the activity delivery model in the community. The purpose of the current study is to identify factors impacting the RTT waiting times across England and their recovery post the COVID-19 pandemic, which could inform future policymaking. Utilising publicly available data from NHS England, we explored the regional RTT variations, as well as the impact of ethnicity and socioeconomic deprivation.

## Methods

### Data sources

Publicly available data sources were used, stored in open access domains in the NHS England website. National data was pooled and broken down by Integrated Care Board (ICB) and commissioning region, aiming to capture the variation in the RTT and respective population characteristics across distinct geographical areas.

We reviewed a period of five years, from August 2019 to August 2024 for commissioning regions, aiming to capture a snapshot of the impact of COVID-19 pandemic and the services’ recovery on regional level and four years, from August 2021 to August 2024 on ICB level. Although Integrated Care Systems existed since 2016, they predominantly operated as informal partnerships, becoming legal entities after the 2022 Health and Care Act [18] and the data of their respective ICBs was made publicly available via NHS England, from 2021.

The dataset that was used for the analysis later described in this manuscript can be found in the RTT Waiting Times data in NHS England website: https://www.england.nhs.uk/statistics/statistical-work-areas/rtt-waiting-times/. Data on deprivation levels was obtained from the 2019 UK Government and Ministry of Housing data on Indices of Multiple Deprivation (IMDs) and the Health Foundation’s data on the deprivation distribution across ICBs. The respective datasets can be accessed through the following links: https://www.gov.uk/government/statistics/english-indices-of-deprivation-2019 and https://www.health.org.uk/reports-and-analysis/briefings/integrated-care-systems-what-do-they-look-like. Lastly, data was analysed on Ethnic Category Coverage per ICB and population per region, available through NHS England: https://digital.nhs.uk/data-and-information/publications/statistical/mi-ethnic-category-coverage/current. A table with their compiled data can be found in Table 1.

**Table 1:** Population, socioeconomic and waiting list characteristics of ICBs.

Integrated Care Board	Population*	Proportion of known ethnicity*	Proportion of white-British patients*	Percentage of LSOAs in the most deprived quintile **	Mean IMD score**	Percentage of patients seen within 18weeks in 2021	Median time in 2021	Proportion of change in patients seen within 18 weeks from 2021 to 2024	Proportion of change in median time from 2021 to 2024
NHS Bath and North East Somerset, Swindon and Wiltshire	1207810	96.02%	80.19%	6.16%	14.39	57.0%	15.5	−4.89%	5.15%
NHS Bedfordshire, Luton and Milton Keynes	1399390	95.69%	54.07%	12.66%	17.95	57.4%	15.3	−18.25%	26.84%
NHS Birmingham and Solihull	2034790	96.93%	42.30%	49.42%	34.17	35.6%	27.7	17.39%	−22.06%
NHS Black Country	1649785	97.67%	56.59%	45.65%	29.88	69.5%	11.9	−32.58%	62.39%
NHS Bristol, North Somerset and South Gloucestershire	1434340	95.65%	72.57%	16.87%	19.47	43.6%	24.5	57.98%	−55.26%
NHS Buckinghamshire, Oxfordshire and Berkshire West	2528460	95.56%	65.74%	2.87%	11.16	48.0%	19.2	14.88%	−17.43%
NHS Cambridgeshire and Peterborough	1371115	95.35%	63.50%	12.45%	16.82	47.5%	19.4	6.87%	−9.19%
NHS Cheshire and Merseyside	3342640	97.54%	81.61%	34.89%	28.24	60.9%	14.2	−21.68%	33.87%
NHS Cornwall and The Isles Of Scilly	707480	97.02%	89.34%	13.15%	23.29	80.2%	9.3	−13.12%	20.42%
NHS Coventry and Warwickshire	1413285	96.64%	61.03%	13.48%	19.15	52.6%	16.7	−15.14%	22.11%
NHS Derby and Derbyshire	1328330	96.87%	81.99%	17.91%	20.08	63.8%	12.9	−24.94%	44.93%
NHS Devon	1545495	96.56%	87.46%	12.59%	20.22	62.8%	13.3	−17.19%	28.56%
NHS Dorset	994030	95.38%	82.36%	8.19%	16.78	44.5%	21.0	5.42%	−7.94%
NHS Frimley	1060220	97.34%	58.11%	2.27%	12.11	66.7%	12.0	−39.79%	92.86%
NHS Gloucestershire	840700	97.03%	83.31%	8.31%	15.06	64.0%	13.9	−35.98%	70.63%
NHS Greater Manchester	4004625	96.84%	61.49%	37.90%	29.30	51.3%	17.3	−12.51%	16.19%
NHS Hampshire and Isle Of Wight	2446835	96.00%	78.49%	11.16%	17.28	60.5%	13.7	−19.27%	35.90%
NHS Herefordshire and Worcestershire	986675	97.31%	82.91%	11.46%	18.31	52.3%	17.3	−17.88%	22.19%
NHS Hertfordshire and West Essex	1987210	96.23%	67.25%	1.87%	13.31	63.1%	13.4	−15.94%	25.11%
NHS Humber and North Yorkshire	2168650	96.26%	84.25%	18.68%	21.24	54.0%	16.3	−15.00%	21.35%
NHS Kent and Medway	2499215	96.97%	76.45%	15.87%	20.16	51.7%	17.3	−10.62%	13.62%
NHS Lancashire and South Cumbria	2190385	97.16%	79.42%	29.68%	26.35	65.8%	11.9	−6.42%	16.88%
NHS Leicester, Leicestershire and Rutland	1539445	96.49%	59.01%	12.27%	18.14	43.9%	21.3	11.08%	−13.34%
NHS Lincolnshire	968705	96.81%	83.80%	15.48%	20.43	49.4%	18.3	−22.97%	33.50%
NHS Mid and South Essex	1517350	96.07%	76.55%	9.79%	16.98	65.7%	13.4	−39.07%	65.31%
NHS Norfolk and Waveney	1311865	96.89%	84.70%	15.71%	21.70	44.9%	21.4	5.66%	−11.48%
NHS North Central London	2371225	96.58%	27.71%	20.63%	22.74	49.2%	18.4	5.33%	−6.63%
NHS North East and North Cumbria	3802480	97.46%	85.09%	32.51%	27.45	75.2%	10.5	−28.37%	55.10%
NHS North East London	3322700	97.86%	24.31%	24.54%	25.56	56.5%	14.5	−1.03%	7.53%
NHS North West London	3897370	94.34%	20.71%	12.84%	20.92	62.6%	14.2	−22.38%	31.02%
NHS Northamptonshire	1009605	96.28%	69.13%	14.69%	18.80	78.7%	8.9	−18.57%	42.86%
NHS Nottingham and Nottinghamshire	1588715	96.59%	71.42%	26.95%	23.54	58.7%	14.1	−27.06%	55.85%
NHS Shropshire, Telford and Wrekin	637870	96.95%	83.32%	12.29%	20.25	68.7%	12.3	−51.26%	110.42%
NHS Somerset	736790	96.11%	86.39%	8.87%	18.70	52.6%	16.7	0.80%	0.03%
NHS South East London	2802935	97.23%	40.59%	17.18%	22.04	58.0%	15.3	−22.60%	32.81%
NHS South West London	2331665	96.62%	41.25%	6.88%	15.65	72.7%	10.7	−18.82%	40.86%
NHS South Yorkshire	1857235	97.00%	75.72%	36.69%	28.67	68.1%	12.1	−24.51%	43.41%
NHS Staffordshire and Stoke-on-Trent	1401545	97.97%	81.14%	19.07%	20.70	56.6%	15.0	−8.69%	16.16%
NHS Suffolk and North East Essex	1287320	97.49%	79.93%	12.46%	19.24	64.4%	12.6	−18.42%	35.65%
NHS Surrey Heartlands	1455450	96.69%	69.18%	0.65%	10.29	75.3%	10.7	−26.58%	49.03%
NHS Sussex	2327810	95.40%	77.71%	9.45%	17.27	66.8%	12.2	−49.04%	117.90%
NHS West Yorkshire	3264025	97.23%	65.79%	35.14%	27.93	57.6%	14.7	−4.03%	7.11%

* 2024/2025 data.

** 2022 data.

#### Definitions.

Throughout the current manuscript there are several terms which may be unfamiliar to service users and experts outside the UK and the NHS. We are compiling a list in this section to facilitate the communication of our findings to a wider audience.

ICBs are the 42 statutory bodies responsible for the planning and funding of most of the NHS activities within England [19]. Commissioning regions are 7 larger geographical areas consisting of a group of ICBs, under the same operational support and supervision [20]. The term “RTT pathway” refers to the first presentation of a referral from primary care (such as a general practitioner) to a consultant in a secondary care (hospital) service for treatment [21]. Any follow-up appointments or re-presentation immediately after discharge do not contribute to the RTT numbers. If a patient is being referred to different services, they will be treated as different entries in the number of patients in the waiting list. In order to mitigate the multiple patient entries, throughout this manuscript we study the impact on the percentage of patients, rather than their absolute numbers.

Deprivation was assessed using the indices of multiple deprivation (IMD) [22] which is the official measure of relative deprivation for small areas in England – the Lower Layer Super Output Area (LSOA)- consisting of about 1,500 residents [23]. Different factors are assessed across the following seven domains: i. income, ii. employment, iii. health deprivation and disability, iv. education, skill and training, v. crime, vi. barriers to housing and services, and vii. living environment [24]. This data is often split in five equal groups, reflecting from the least deprived to the most deprived areas within a LSOA. Public organisations have often used the deprivation quintiles to report trends or phenomena impacting the most or least deprived areas [25]. Most recently in the national NHS approach for addressing healthcare inequalities known as ‘CORE20PLUS5’, the ‘CORE20’ referred to is the most deprived 20% or quintile of a population.

The term “ethnic minority” is used to describe the non-“white British” population of England.

### Data analysis

Data was extracted and reviewed independently by two authors. Microsoft Excel and R software (version 4.4.2, R Foundation for Statistical Computing, Vienna, Austria) was used for data synthesis and analysis. Data on population was extracted by ICB and pooled into their respective regional distribution. Deprivation and ethnicity data was extracted by ICB, then analysed and reported at an ICB level. Due to missing data on the ethnic characteristics of the populations per ICB, the analysis for ethnicity was conducted on the percentage of white-British patients amidst the patients with known ethnicity.

Data for the waiting times was reported in median waiting in weeks, while data on the number of patients seen within the target of 18 weeks was reported as a percentage of the total of the referrals received by the specific ICB/region. IMD score and percentage of LSOAs in the most deprived quintile were handled as a continuous measure per ICB.

The data was not assumed to be normally distributed, as such Spearman test was used for the analysis. Correlation was assessed on ICB level between each of the variables of population, ethnic minority background and deprivation, and the waiting times in the first capture point, the percentage of patients seen within the 18-weeks, the change of waiting times over time, as well as the change in percentage of patients seen within 18-weeks over time. Bonferroni correction would be applied for statistically significant results.

## Results

**Fig 1:**
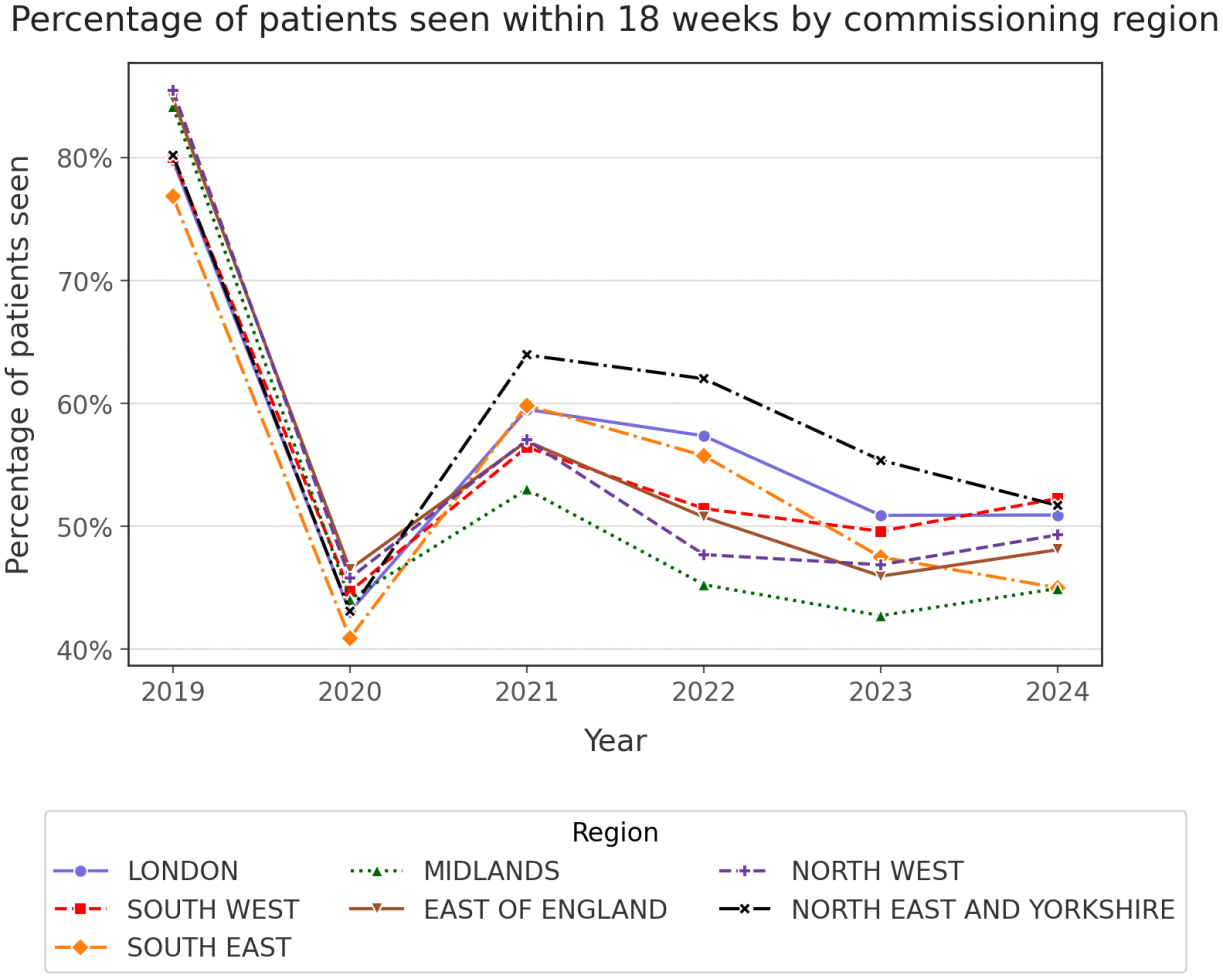
Percentage of patients treated within 18 weeks by commissioning region.

**Fig 2:**
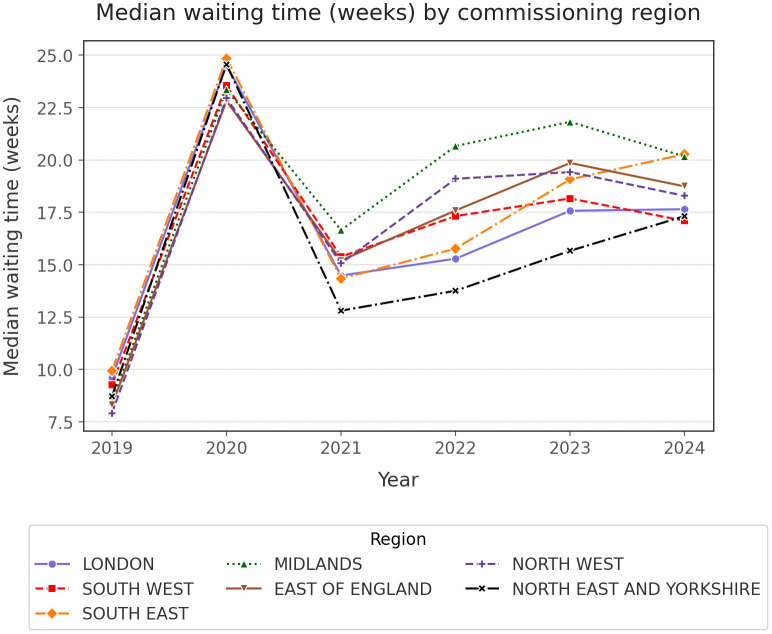
Median waiting time (weeks) by commissioning region.

On a regional level, over the course of the last five years, an increase was noted across all commissioning groups, some of which experienced a doubling of the number of patients waiting for secondary care appointment (Supplementary Table 1, 2). Amongst commissioning regions, the Midlands demonstrated the highest number of patients in waiting presenting a 57.8% increase within five years, while at the other end of the spectrum, South-West had the lowest number of patients, experiencing a 44% increase in the past five years. With respect to the percentage of patients seen within the constitutional NHS target, a decline has been noted across all regions (Fig 1). While a short recovery period was noted in 2021, the RTT waiting list numbers have demonstrated a steady decline over the course of 2022–2024. Median waiting time has been mirroring this trend, demonstrating a sharp increase during the onset of the COVID-19 pandemic and a steady increase thereafter, after a short recovery in 2021 (Fig 2).

**Fig 3:**
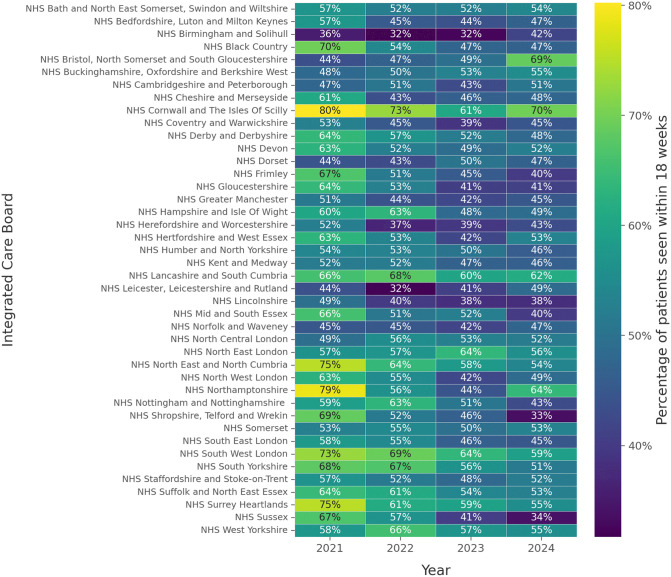
Heat map of the percentage of patients treated within 18 weeks per ICB, per year.

**Fig 4:**
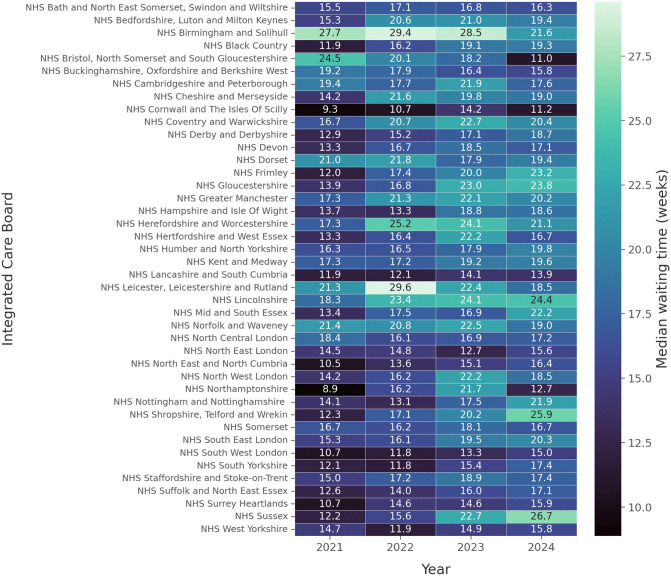
Heat map of the median waiting time in weeks per ICB, per year.

NHS Cornwall and the Isles of Scilly ICB managed to meet the NHS standard more frequently than other ICBs in 2021, 2022 and 2024 (80.2%, 72.8% and 69.7% respectively), while South West London and North East London ICBs outperformed their counterparts in 2023 (63.2% each) (Supplementary Table 3). NHS Birmingham and Solihull ICB demonstrated the lowest percentage of patients that were seen within 18 weeks in 2021, 2022 and 2023 (35.6%, 31.7% and 32.5% respectively), while NHS Shropshire, Telford and Wrekin ICB demonstrated the smallest proportion for 2024 (33.5%) (Fig 3). The variability across ICBs’ performance is mirrored in their respective median waiting times, with ICBs with larger proportions of patients treated within 18 weeks also demonstrating lower median waiting times compared to worse performing ICBs (Fig 4).

### Population

Spearman’s correlation drawn between the population of each ICB and percentage of patients seen in 2021, as well as the waiting time at that point, demonstrated no significant association (ρ = 0.05, p = 0.77 and ρ = 0.01, p = 0.96, respectively). Furthermore, there was no association between the size of the population of an ICB and the change in the percentage of patients seen over the period 2021–2024 (ρ = 0.07, p = 0.67) or the change in the waiting times during that period (ρ = −0.08, p = 0.6) (Table 2).

**Table 2:** Spearman correlation testing results of population size, deprivation and ethnic characteristics on waiting lists.

	Population	Proportion of white-British patients	Mean IMD score	Percentage of LSOAs in the most deprived quintile
Percentage of patients seen within 18weeks in 2021	ρ = 0.05, p = 0.77	ρ = 0.13, p = 0.43	ρ = −0.11, p = 0.48	ρ = −0.11, p = 0.51
Median time in 2021	ρ = 0.01, p = 0.96	ρ = −0.11, p = 0.48	ρ = 0.07, p = 0.65	ρ = 0.07, p = 0.65
Proportion of change in patients seen within 18 weeks from 2021 to 2024	ρ = 0.07, p = 0.67	ρ = 0.11, p = 0.47	ρ = 0.1, p = 0.51	ρ = 0.10, p = 0.51
Proportion of change in median time from 2021 to 2024	ρ = −0.08, p = 0.6	ρ = 0.11, p = 0.48	ρ = −0.12, p = 0.45	ρ = −0.12, p = 0.45

### Ethnicity

Ethnicity’s impact on waiting times and percentage of patients seen within 18 weeks was assessed on an ICB level (Table 2). Using Spearman’s rank correlation, the percentage of white-British patients was correlated to the percentage of patients seen within 18 weeks in 2021, demonstrating a weak positive but not statistically significant association (ρ = 0.13, p = 0.43), and the median waiting time in 2021, revealing a weak negative but not significant association (ρ = −0.11, p = 0.48). There was also not statistically significant evidence of monotonic relationship between the “white-British” ethnic group and the changes in proportion of patients seen within 18 weeks from 2021 to 2024 (ρ = 0.11, p = 0.47) or the changes in the median waiting times (ρ = 0.11, p = 0.48).

### Deprivation

Deprivation data was assessed by IMD score (where higher score means higher deprivation) and percentage of most deprived quintile of LSOAs, per ICB (Table 2). Spearman’s rank correlation was undertaken to assess any relationship between the IMD score and the percentage of patients seen within 18 weeks in 2021 (ρ = −0.11, p = 0.48), as well as the median waiting time (ρ = 0.07, p = 0.65), revealing an absence of statistically significant monotonic association. There was no evidence of a statistically significant association between the deprivation and the changes in proportion of patients seen within 18 weeks from 2021 to 2024 (ρ = 0.1, p = 0.51) or the changes in the median waiting times (ρ = −0.12, p = 0.45).

Spearman’s test was also undertaken for the percentage of most deprived LSOAs in regards to the same variables as IMD score, demonstrating no evidence of statistically significant association. Specifically, the respective test values are the following: ρ = −0.11, p = 0.51 for the percentage of patients seen within 18 weeks, ρ = 0.07, p = 0.65 for the median waiting time, ρ = 0.10, p = 0.51 for the changes in proportion of patients seen within 18 weeks and ρ = −0.12, p = 0.45 for the changes in the median waiting times.

## Discussion

In this manuscript, the status of the waiting lists across the different ICBs and commissioning regions of England was reviewed, from the later stages of the pandemic, and their respective recovery. Waiting times and capacity of patients reviewed has been recovering after the pandemic, but the recovery curve has flattened after 2022 (Figs 1, 2). When the same data was reviewed on the ICB level, significant variability was found amongst the different ICBs, with improving or deteriorating RTT metrics on an annual basis (Figs 3, 4). A compilation of factors that could contribute to their variation, including the differences of population size, ethnicity and deprivation, were analysed revealing no statistically significant association.

### Deprivation

These results do not support the absence of association between deprivation and healthcare disparities in general, but suggest that on an ICB level this relationship is not apparent. A potential interpretation of this may be attributed to the structure of the ICBs, consisting of multiple LSOAs with different deprivation characteristics, resulting in more homogenous areas compared to socioeconomic variability across NHS Trusts. This can be postulated after reviewing the deprivation data of the ICBs: comparing Surrey Heartlands ICB, which had 0.7% of the more deprived quintile of LSOAs, to Birmingham and Solihull ICB (49.4% of the more deprived quintile), their respective IMD score difference was −23.9, which does not capture the significant difference between the least and the most deprived ICBs. A different interpretation may arise from the nature of the scoring itself- IMD scores and quintiles of deprivation are relative measures, predominantly facilitating the ranking of different areas, without reflecting the absolute deprivation of an area. Different authors have argued about the validity of composite deprivation markers capturing the true effect of a specific socioeconomic phenomenon- such as education, employment or rurality- to healthcare access [7,26]. From a population health perspective, it is a well acknowledged problem that aggregating up over larger, heterogenous geographical areas can mask significant variation in health status, with pockets of deprivation existing within larger, more affluent areas. This understanding is one of the driving factors behind the move to Neighbourhood Health Services; a new way of more integrated, place-based working for the NHS (of which the elective care reform plan is an example). The complexities of analysis of deprivation over large geographies as discussed in this paper add further weight to the argument for place-based care- the idea that devolving budgets and decision-making to a more local level allows an integrated, partnership approach which is organised around local geogrpahies and better able to respond to local need [27,28].

Although a correlation between the socioeconomic status and the effect of the COVID-19 pandemic was not established on the level of ICB areas, there was variability in the waiting times and the proportion of patients seen, as illustrated in Fig 3 and 4. Organisations, such as the King’s Fund, having studied the early impact of COVID-19 on healthcare, reported that individuals in the most deprived areas were 60% more likely to experience prolonged waits (>52 weeks) than those in affluent regions, with Northern England exhibiting the longest delays [29], between 2020 and 2021. A detailed breakdown and investigation by the King’s Fund, during the peak of the pandemic, revealed that waiting times increased by 55.2% in areas with the highest IMD, compared to 36% in the least deprived areas [30]. Similarly, The Nuffield Trust and Health Foundation reported 21% of patients’ waiting time exceeding one year across the most deprived areas, compared to 12% of patients in the least deprived areas [31].

While the IMD score and lowest quintile of LSOAs, did not reflect such a relationship, we recognise a potential limitation of this study, where such an analysis on the more homogeneous level of NHS Trusts, may have yielded a stronger association. Although such an analysis exceeds the scope of this manuscript, correlation and regression model have been recognised to differ according to the scale and size of the spatial reporting units [32–34]. Another challenge recognised relating to the scalability of spatial models, concerns the uncertainty caused by the different delineation of analysis units, as described by Kwan (2012) [35].

### Ethnicity

Ethnicity was recognised as one of the variables affecting access to healthcare across the literature; being from a non-“white British” ethnic background is recognised as one of the factors predisposing to health inequalities, including worsening waiting times in both elective and emergency care [ 31,36,37]. Prolonged waiting times have been shown to have a detrimental impact on patients [38] and particularly their mental health and quality of life [39]. Delayed appointments also increase healthcare costs. Waiting times have been described as an indicator for a health service under strain [40], underlining the importance of tackling the complex social circumstances that burden such services, such as poverty [41]. The ethnographic study of the Taskforce of Multiple Conditions [42], highlighted the intersectionality of such disadvantages. Patients of non-white ethnic backgrounds, who were living in an area of socioeconomic deprivation were more likely to suffer from the summative effects of adverse outcomes, including limited access to healthcare or poor compliance to treatment regimens [42].

Several authors have worked on identifying disparities of access to emergency healthcare by ethnic minority groups [43–46], providing compelling evidence on inequalities varying from waiting times in the emergency department to simple pain relief. There is, however, limited evidence on the impact of ethnicity on the referral pathways for elective care. Fluck et al (2025) recently studied elective admissions to the hospital in the UK [47], reviewing data of a single NHS Trust in Surrey from 2019 to 2023, demonstrating a strong correlation between prolonged waiting, and ethnic minority background and deprivation. An older study by Morgan et al (2004), assessing the waiting times in a single hospital in London, suggested that there was a reverse correlation with the white-British experiencing longer waiting times [48]. This study focuses on the RTT waiting times to the first appointment, and throughout our analysis there was not evidence of correlation between ethnicity and waiting time. While this may be attributed to a masking effect by the diverse LSOAs of ICBs, the discrepancies have been predominantly reported on the admission pathways of the hospital rather than the waiting times, offering an alternative interpretation that the RTT waiting times are not as impacted by ethnicity as the emergency and routine admissions.

### ENT practice

Currently in England, ENT is one of the most heavily affected specialties in regards to the RTT waiting times [49]. According to the Getting It Right First Time (GIRFT) update in 2023, a threefold increase was noted over the last 10 years, with current numbers exceeding 700,000 patients [50]. This growth may indicate systemic inefficiencies and capacity constraints, exacerbated by the COVID-19 pandemic, which redirected healthcare resources and reduced elective service capacity [16]. Given the high incidence of ENT-related symptoms in the general population [51], the challenges of RTT waiting lists are further aggravated for the ENT services.

The increased demand was partially mitigated by an increase in the capacity, NHS staff delivering services at a 5% increased capacity compared to 2019, treating 18 million patients in 2024 [52]. Unfortunately, as we highlighted earlier, through the plateauing of the recovery curves in Fig 1 and 2, such measures did not resolve the continued increase in the demand and consequently in the waiting times. The previous and current governments have sought a partnership with the independent sector, where patients can be outsourced, increasing further the capacity of the NHS [53]. Such attempts to mitigate some of the RTT waiting times burden, started before the COVID-19 pandemic [54], but now have become widely accepted as a mainstay strategy by the stakeholders. There is, however, scepticism on whether the involvement of private sector would reduce the gap of accessibility to healthcare for the underserved populations, which is now transforming the landscape of healthcare into a more complex environment to navigate, demanding more information from the service users, in order to reach any decision [55]. The concerns on private sector being a hidden driving force behind health inequalities were explored by Kirkwood et al (2025) [56], who demonstrated an increase in health disparities and in the RTT waiting times, upon the introduction of the private sector in healthcare in 2003.

In January 2025, NHS England released their new strategy on reforming elective care for patients, highlighting the importance of a paradigm shift away from hospital care [17]. Identifying that over 80% of elective pathways conclude without patients being admitted, the focus is now the digital transformation of the service and patient’s choice and accessibility. ENT was identified as one of the five specialties that need to set clear targets and transform the activity delivery model in the community [17]. Early attempts of implementing community-based services have been reported by different teams. Cottrell et al (2020) [ 57] sought to implement a primary-care based triaging tool via endoscopic video capture, along with clinical examination by a general practitioner and hearing test, reporting success in their pilot. A slightly different approach with a community-based audiology service, has been launched by the ENT team of the University College London Hospitals [58], but there are not any preliminary results available on the performance of their pathway. Remote otology and rhinology services both within England, and beyond, have been reported to delivery equitable healthcare service and tackling accessibility restrictions for the hardly reached, underserved communities, while at the same time increasing consultant capacity to review a higher volume of patients [59]. Further investigation is needed to assess whether such digitally-enabled services may actually play a role in improving the RTT waiting times for ENT, as concerns have been voiced on the technological access of the more deprived communities, potentially increasing the socioeconomic disparity in healthcare access [60,61].

## Conclusion

ENT services across England have experienced significant pressures that have not been alleviated after the conclusion of the COVID-19 pandemic. This reflects RTT waiting times worsening over the past five years with clear regional disparities persisting post-pandemic. Despite clear evidence from other sources on the impact of deprivation on access to healthcare, this association was not demonstrated clearly through our analysis, suggesting further confounding or masked variation of waiting times across different areas of England. The shift in NHS policy towards community-based care models, represents a novel opportunity for ENT, as a specialty with a high proportion of non-surgical management pathways, being uniquely positioned to benefit from this transformation.

## Supporting information

S1 Table Percentage of patients seen within 18 weeks per year per region. (DOCX)

S2 Table Median waiting time (weeks) per year per region. (DOCX)

S3 Table Percentage of patients seen within 18 weeks per year per ICB. (DOCX)
