# Supplementary material for: Referral to treatment times in the National Health Service of England: A five-year analysis of the impact of the COVID-19 Pandemic and socioeconomic deprivation and future implications for Ear, Nose and Throat service delivery
Source: PLoS One. 2026 Apr 6;21(4):e0346596. doi: 10.1371/journal.pone.0346596 (PMC13052864; doi:10.1371/journal.pone.0346596)
Supplement: S1 Table — (DOCX) [file pone.0346596.s001.docx]

**Supplementary Table 1: Percentage of patients seen within 18 weeks per year per region**

|  | **Percentage within 18 weeks** | | | | | |
| --- | --- | --- | --- | --- | --- | --- |
|  | 2019 | 2020 | 2021 | 2022 | 2023 | 2024 |
| LONDON COMMISSIONING REGION | 79.8% | 43.1% | 59.5% | 57.4% | 50.9% | 50.9% |
| SOUTH WEST COMMISSIONING REGION | 80.0% | 44.7% | 56.4% | 51.5% | 49.6% | 52.3% |
| SOUTH EAST COMMISSIONING REGION | 76.8% | 40.9% | 59.9% | 55.7% | 47.5% | 45.0% |
| MIDLANDS COMMISSIONING REGION | 84.2% | 44.1% | 53.0% | 45.3% | 42.7% | 45.0% |
| EAST OF ENGLAND COMMISSIONING REGION | 84.8% | 46.5% | 56.9% | 50.8% | 46.0% | 48.1% |
| NORTH WEST COMMISSIONING REGION | 85.5% | 45.8% | 57.0% | 47.7% | 46.9% | 49.3% |
| NORTH EAST AND YORKSHIRE COMMISSIONING REGION | 80.2% | 43.1% | 63.9% | 62.0% | 55.4% | 51.7% |
