# Supplementary material for: Referral to treatment times in the National Health Service of England: A five-year analysis of the impact of the COVID-19 Pandemic and socioeconomic deprivation and future implications for Ear, Nose and Throat service delivery
Source: PLoS One. 2026 Apr 6;21(4):e0346596. doi: 10.1371/journal.pone.0346596 (PMC13052864; doi:10.1371/journal.pone.0346596)
Supplement: S2 Table — (DOCX) [file pone.0346596.s002.docx]

**Supplementary Table 2: Median waiting time (weeks) per year per region**

|  | **Median waiting time (in weeks)** | | | | | |
| --- | --- | --- | --- | --- | --- | --- |
|  | 2019 | 2020 | 2021 | 2022 | 2023 | 2024 |
| LONDON COMMISSIONING REGION | 9.6 | 24.5 | 14.5 | 15.3 | 17.6 | 17.6 |
| SOUTH WEST COMMISSIONING REGION | 9.3 | 23.5 | 15.4 | 17.3 | 18.2 | 17.1 |
| SOUTH EAST COMMISSIONING REGION | 9.9 | 24.8 | 14.3 | 15.8 | 19.1 | 20.3 |
| MIDLANDS COMMISSIONING REGION | 8.5 | 23.4 | 16.7 | 20.7 | 21.8 | 20.2 |
| EAST OF ENGLAND COMMISSIONING REGION | 8.3 | 22.8 | 15.2 | 17.6 | 19.9 | 18.7 |
| NORTH WEST COMMISSIONING REGION | 7.9 | 22.9 | 15.1 | 19.1 | 19.4 | 18.3 |
| NORTH EAST AND YORKSHIRE COMMISSIONING REGION | 8.7 | 24.6 | 12.8 | 13.8 | 15.7 | 17.3 |
