# Supplementary material for: Referral to treatment times in the National Health Service of England: A five-year analysis of the impact of the COVID-19 Pandemic and socioeconomic deprivation and future implications for Ear, Nose and Throat service delivery
Source: PLoS One. 2026 Apr 6;21(4):e0346596. doi: 10.1371/journal.pone.0346596 (PMC13052864; doi:10.1371/journal.pone.0346596)
Supplement: S3 Table — (DOCX) [file pone.0346596.s003.docx]

**Supplementary Table 3: Percentage of patients seen within 18 weeks per year per ICB**

|  | **Median waiting time (in weeks)** | | | | |
| --- | --- | --- | --- | --- | --- |
|  | 2021 | 2022 | 2023 | 2024 | change in waiting time (2024-2021) |
| NHS BATH AND NORTH EAST SOMERSET, SWINDON AND WILTSHIRE INTEGRATED CARE BOARD | 15.5 | 17.1 | 16.8 | 16.3 | 5.147876362 |
| NHS BEDFORDSHIRE, LUTON AND MILTON KEYNES INTEGRATED CARE BOARD | 15.3 | 20.6 | 21.0 | 19.4 | 26.84047229 |
| NHS BIRMINGHAM AND SOLIHULL INTEGRATED CARE BOARD | 27.7 | 29.4 | 28.5 | 21.6 | -22.05953968 |
| NHS BLACK COUNTRY INTEGRATED CARE BOARD | 11.9 | 16.2 | 19.1 | 19.3 | 62.39429726 |
| NHS BRISTOL, NORTH SOMERSET AND SOUTH GLOUCESTERSHIRE INTEGRATED CARE BOARD | 24.5 | 20.1 | 18.2 | 11.0 | -55.25902669 |
| NHS BUCKINGHAMSHIRE, OXFORDSHIRE AND BERKSHIRE WEST INTEGRATED CARE BOARD | 19.2 | 17.9 | 16.4 | 15.8 | -17.43183997 |
| NHS CAMBRIDGESHIRE AND PETERBOROUGH INTEGRATED CARE BOARD | 19.4 | 17.7 | 21.9 | 17.6 | -9.190037334 |
| NHS CHESHIRE AND MERSEYSIDE INTEGRATED CARE BOARD | 14.2 | 21.6 | 19.8 | 19.0 | 33.86750975 |
| NHS CORNWALL AND THE ISLES OF SCILLY INTEGRATED CARE BOARD | 9.3 | 10.7 | 14.2 | 11.2 | 20.4224 |
| NHS COVENTRY AND WARWICKSHIRE INTEGRATED CARE BOARD | 16.7 | 20.7 | 22.7 | 20.4 | 22.11024607 |
| NHS DERBY AND DERBYSHIRE INTEGRATED CARE BOARD | 12.9 | 15.2 | 17.1 | 18.7 | 44.92583346 |
| NHS DEVON INTEGRATED CARE BOARD | 13.3 | 16.7 | 18.5 | 17.1 | 28.5633251 |
| NHS DORSET INTEGRATED CARE BOARD | 21.0 | 21.8 | 17.9 | 19.4 | -7.936550749 |
| NHS FRIMLEY INTEGRATED CARE BOARD | 12.0 | 17.4 | 20.0 | 23.2 | 92.85592007 |
| NHS GLOUCESTERSHIRE INTEGRATED CARE BOARD | 13.9 | 16.8 | 23.0 | 23.8 | 70.62550937 |
| NHS GREATER MANCHESTER INTEGRATED CARE BOARD | 17.3 | 21.3 | 22.1 | 20.2 | 16.18626236 |
| NHS HAMPSHIRE AND ISLE OF WIGHT INTEGRATED CARE BOARD | 13.7 | 13.3 | 18.8 | 18.6 | 35.90265865 |
| NHS HEREFORDSHIRE AND WORCESTERSHIRE INTEGRATED CARE BOARD | 17.3 | 25.2 | 24.1 | 21.1 | 22.19415577 |
| NHS HERTFORDSHIRE AND WEST ESSEX INTEGRATED CARE BOARD | 13.4 | 16.4 | 22.2 | 16.7 | 25.1093319 |
| NHS HUMBER AND NORTH YORKSHIRE INTEGRATED CARE BOARD | 16.3 | 16.5 | 17.9 | 19.8 | 21.34710583 |
| NHS KENT AND MEDWAY INTEGRATED CARE BOARD | 17.3 | 17.2 | 19.2 | 19.6 | 13.62382609 |
| NHS LANCASHIRE AND SOUTH CUMBRIA INTEGRATED CARE BOARD | 11.9 | 12.1 | 14.1 | 13.9 | 16.87747497 |
| NHS LEICESTER, LEICESTERSHIRE AND RUTLAND INTEGRATED CARE BOARD | 21.3 | 29.6 | 22.4 | 18.5 | -13.33872272 |
| NHS LINCOLNSHIRE INTEGRATED CARE BOARD | 18.3 | 23.4 | 24.1 | 24.4 | 33.49566776 |
| NHS MID AND SOUTH ESSEX INTEGRATED CARE BOARD | 13.4 | 17.5 | 16.9 | 22.2 | 65.3067135 |
| NHS NORFOLK AND WAVENEY INTEGRATED CARE BOARD | 21.4 | 20.8 | 22.5 | 19.0 | -11.48149846 |
| NHS NORTH CENTRAL LONDON INTEGRATED CARE BOARD | 18.4 | 16.1 | 16.9 | 17.2 | -6.63492259 |
| NHS NORTH EAST AND NORTH CUMBRIA INTEGRATED CARE BOARD | 10.5 | 13.6 | 15.1 | 16.4 | 55.10122233 |
| NHS NORTH EAST LONDON INTEGRATED CARE BOARD | 14.5 | 14.8 | 12.7 | 15.6 | 7.527437455 |
| NHS NORTH WEST LONDON INTEGRATED CARE BOARD | 14.2 | 16.2 | 22.2 | 18.5 | 31.01596061 |
| NHS NORTHAMPTONSHIRE INTEGRATED CARE BOARD | 8.9 | 16.2 | 21.7 | 12.7 | 42.85700159 |
| NHS NOTTINGHAM AND NOTTINGHAMSHIRE INTEGRATED CARE BOARD | 14.1 | 13.1 | 17.5 | 21.9 | 55.8509211 |
| NHS SHROPSHIRE, TELFORD AND WREKIN INTEGRATED CARE BOARD | 12.3 | 17.1 | 20.2 | 25.9 | 110.4221266 |
| NHS SOMERSET INTEGRATED CARE BOARD | 16.7 | 16.2 | 18.1 | 16.7 | 0.033233632 |
| NHS SOUTH EAST LONDON INTEGRATED CARE BOARD | 15.3 | 16.1 | 19.5 | 20.3 | 32.80941801 |
| NHS SOUTH WEST LONDON INTEGRATED CARE BOARD | 10.7 | 11.8 | 13.3 | 15.0 | 40.86044625 |
| NHS SOUTH YORKSHIRE INTEGRATED CARE BOARD | 12.1 | 11.8 | 15.4 | 17.4 | 43.40850152 |
| NHS STAFFORDSHIRE AND STOKE-ON-TRENT INTEGRATED CARE BOARD | 15.0 | 17.2 | 18.9 | 17.4 | 16.15543475 |
| NHS SUFFOLK AND NORTH EAST ESSEX INTEGRATED CARE BOARD | 12.6 | 14.0 | 16.0 | 17.1 | 35.64526466 |
| NHS SURREY HEARTLANDS INTEGRATED CARE BOARD | 10.7 | 14.6 | 14.6 | 15.9 | 49.0299696 |
| NHS SUSSEX INTEGRATED CARE BOARD | 12.2 | 15.6 | 22.7 | 26.7 | 117.9012252 |
| NHS WEST YORKSHIRE INTEGRATED CARE BOARD | 14.7 | 11.9 | 14.9 | 15.8 | 7.111221884 |
